# Activated Microglia in Cortical White Matter Across Cognitive Aging Trajectories

**DOI:** 10.3389/fnagi.2019.00094

**Published:** 2019-05-14

**Authors:** Tamar Gefen, Garam Kim, Kabriya Bolbolan, Andrew Geoly, Daniel Ohm, Carly Oboudiyat, Ryan Shahidehpour, Alfred Rademaker, Sandra Weintraub, Eileen H. Bigio, M.-Marsel Mesulam, Emily Rogalski, Changiz Geula

**Affiliations:** ^1^Mesulam Center for Cognitive Neurology and Alzheimer’s Disease, Feinberg School of Medicine, Northwestern University, Chicago, IL, United States; ^2^Department of Psychiatry and Behavioral Sciences, Feinberg School of Medicine, Northwestern University, Chicago, IL, United States; ^3^Department of Preventive Medicine, Feinberg School of Medicine, Northwestern University, Chicago, IL, United States; ^4^Department of Pathology, Feinberg School of Medicine, Northwestern University, Chicago, IL, United States; ^5^Department of Neurology, Feinberg School of Medicine, Northwestern University, Chicago, IL, United States; ^6^Department of Cell and Molecular Biology, Feinberg School of Medicine, Northwestern University, Chicago, IL, United States

**Keywords:** microglia, white matter, memory, cognitive aging, neurodegeneration

## Abstract

Activation of microglia, the primary mediators of inflammation in the brain, is a major component of gliosis and neuronal loss in a number of age-related neurodegenerative disorders, such as Alzheimer’s disease (AD). The role of activated microglia in white matter, and its relationship with cognitive decline during aging are unknown. The current study evaluated microglia densities in the white matter of postmortem specimens from cognitively normal young adults, cognitively normal older adults, and cognitive “SuperAgers,” a unique group of individuals over age 80 whose memory test scores are at a level equal to or better than scores of 50-to-65-year-olds. Whole hemisphere sections from cognitively normal old, young, and “SuperAgers” were used to quantify densities of human leukocyte antigen-D related (HLA-DR)-positive activated microglia underlying five cortical regions. Statistical findings showed a significant main effect of group on differences in microglia density where cognitively normal old showed highest densities. No difference between SuperAgers and young specimens were detected. In two autopsied SuperAgers with MRI FLAIR scans available, prominent hyperintensities in periventricular regions were observed, and interestingly, examination of corresponding postmortem sections showed only sparse microglia densities. In conclusion, activated microglia appear to respond to age-related pathologic changes in cortical white matter, and this phenomenon is largely spared in SuperAgers. Findings offer insights into the relationship between white matter neuroinflammatory changes and cognitive integrity during aging.

## Introduction

Microglia are the resident macrophages of the central nervous system (CNS), and dynamically survey their surroundings for foreign antigens, signs of infection, and cell distress (Streit et al., 2014). The precise roles of microglia in brain health and disease are not well understood; while the activation of microglia is usually an initial advantageous response to insult, prolonged activation has been suggested to lead to cytotoxic effects (McGeer and McGeer, 2001; Luo et al., 2010). In age-related neurodegenerative diseases, like Alzheimer’s disease (AD), activated microglia appear to be associated with pathological inclusions, such as amyloid-β plaques and neurofibrillary tangles—the characteristic neuropathologic substrates of AD—within cortical gray matter (Serrano-Pozo et al., 2011). Microglia proliferation and activation appear to increase, not only in response to neurodegenerative pathogens but also in brain specimens of cognitively normal elderly who do not show Alzheimer’s pathology (Conde and Streit, 2006).

The characterization of microglia activation in white matter has received some experimental attention in psychiatric illnesses (Najjar and Pearlman, 2015), developmental or congenital abnormalities (e.g., leukodystrophies, hypoxia-ischemia, etc.; Billiards et al., 2006; van der Knaap and Bugiani, 2017), and neurodegenerative disorders like AD; one recent qualitative study showed an age-dependent increase in microglia activation in postmortem samples of human white matter, with changes detected as early as age ~50 years (Raj et al., 2017). To date, there are no quantitative examinations on regional densities of white matter microglia across cognitive aging trajectories.

The primary purpose of this study was to evaluate microglia densities in the white matter of postmortem specimens from cognitively normal young adults, cognitively normal old adults, and cognitive “SuperAgers” are a unique group of individuals over age 80 whose memory test scores are at a level equal to or better than scores of 50-to-65-year-olds. In a recent investigation, SuperAgers showed low levels of neurofibrillary degeneration in the hippocampal-entorhinal complex mostly in the Braak II–III stages; these regions also showed generally preserved neuronal integrity (Rogalski et al., 2018). We show in this study significant age-related activation of microglia in the white matter of cognitively normal elderly, in contrast to SuperAging specimens, which show limited densities of activated microglia in white matter in areas that underlie limbic and neocortical regions. In SuperAger cases, microglia densities were strikingly similar to individuals 30–40 years their junior. Finally, we highlight two autopsied SuperAger cases with MRI FLAIR scans available during life to evaluate the association between postmortem microglia burden and the degree of white matter hyperintensities (WMHs), a presumed radiographic measure of white matter integrity (Wardlaw et al., 2015).

This investigation of postmortem specimens offers insights into the integrity of white matter in the aging brain and clarifies the contribution of white matter activated microglia across cognitive aging trajectories.

## Materials and Methods

### Case Characterization

Brain tissue was obtained from a number of neuropathologists across the US and from the Northwestern University AD Center Brain Bank. See Table 1 for characteristics of cases. Seventeen cases were identified based on age and cognitive status.

**Table 1:** Case characteristics.

Case #	Group	Age at death (range)	PMI (hours)	Brain weight (gms)
1	Young	45–50	13	1,650
2	Young	20–24	12	1,300
3	Young	55–60	42	1,100
4	Young	45–50	48	1,150
5	Young	55–60	20	1,300
6	Normal Old	85–90	12	1,250
7	Normal Old	80–84	24	NA
8	Normal Old	85–90	6	1,160
9	Normal Old	95–100	5	NA
10	Normal Old	70–74	16	1,600
11	Normal Old	75–80	42	1,200
12	Normal Old	70–74	13	1,150
13	SuperAger	85–90	5	1,100
14	SuperAger	85–90	11	1,090
15	SuperAger	85–90	4	990
16	SuperAger	85–90	NA	1,240
17	SuperAger	95–100	58	1,020

*PMI, Post-mortem interval; gms, grams; NA, not available*.

Of the 17 cases, five were considered to be “cognitively normal young” (“young”), seven cases were designated as “cognitively normal elderly” (“normal old”), and a third group of brain specimens were obtained from cognitive SuperAgers based on criteria described below. All cases were native English speakers, fully independent in activities of daily living, and visual acuity was required to be at least 20/30. Hearing was required to be adequate for conversation. Cases had to show an absence of significant antemortem neurological or psychiatric history. This includes a history of CNS disease, DSM-5 criteria for any major psychiatric disorder, alcohol or substance abuse, or chronic use of neuroleptic or hypnotic medications. All SuperAgers and Cases 6, 8, 9 underwent neuropsychological testing. Cases identified as cognitively normal young and old cases without neuropsychological testing were based on clinical review of records. Information was obtained from the next of kin whenever necessary. There was no indication of prior autoimmune disease in any of the cases; one case (Case 1, age 45) died of non-Hodgkin’s lymphoma. Two SuperAging cases had MRI FLAIR scans available prior to death. The study was approved by the Northwestern University Institutional Review Board and conducted in accordance with the Helsinki Declaration^1^. Written informed consent was obtained for the collection of human tissue.

### Criteria for Cognitive SuperAgers and Cognitively Normal Elderly

Cognitive SuperAgers represent individuals whose performance on tests of episodic memory is at least as good as individuals 20–30 years their junior. Thus, SuperAgers appear to have resisted the downward trend of cognitive decline that is considered “typical” with advancing age. SuperAgers were identified based on their age (at least 80-years-old at the time of enrollment) and were required to meet strict psychometric criteria on a battery of neuropsychological tests, which were chosen for their relevance to cognitive aging and their sensitivity to detect clinical symptoms associated with AD (Gefen et al., 2014). Briefly, the delayed recall score of the Rey Auditory Verbal Learning Test (RAVLT; Schmidt, 2004) was used as a measure of episodic memory and SuperAgers were required to perform at or above average normative values for individuals in their 50s and 60s (midpoint age = 61; RAVLT delayed-recall raw score ≥9; RAVLT delayed-recall scaled score ≥10). A 30-item version of the Boston Naming Test (“BNT-30”; Kaplan et al., 2001), Trail-Making Test (TMT) Part B (Reitan, 1958), and Category Fluency test (“Animals”; Morris et al., 1989) were used to measure cognitive function in non-memory domains. On the BNT-30, TMT Part B, and Category Fluency, SuperAgers were required to perform within or above one standard deviation of the average range according to published normative values based on age and demographic factors (Saxton et al., 2000; Shirk et al., 2011). Cognitively normal elderly that had undergone neuropsychological evaluation were required to perform within one standard deviation of the average range for their age and education according to published normative values on the RAVLT, BNT-30, TMT Part B, and Category Fluency measures.

### Tissue Preparation and Quantitation

All brains were examined macroscopically and microscopically by a neuropathologist (EB). There was no major vascular disease and no evidence of major neurodegeneration other than Alzheimer-type neurofibrillary tangles and amyloid deposits, which were attributed to age. Brains were fixed in either formalin or 4% paraformaldehyde. A 1-in-24 series of 40 μm-thick whole hemisphere coronal sections was collected from each of the following regions (5–6 sections per region): corpus callosum (CC), anterior cingulate cortex (ACC), inferior frontal gyrus (IFG), superior temporal gyrus (STG), inferior parietal lobule (IPL), and the entorhinal-hippocampus complex (ERC). Activated microglia were visualized immunohistochemically using an antibody against the human leukocyte antigen-D related (HLA-DR) protein (mouse monoclonal; Dako; 1/1000). HLA-DR is a cell surface class II glycoprotein of the major histocompatibility complex that mediates the presentation of foreign antigens in normal and diseased brains (De Tribolet et al., 1984; McGeer et al., 1988; Gehrmann et al., 1993). A subset of cases (2, 10, 16) were immunostained with ionized calcium binding adaptor molecule 1 (Iba1) to visualize resting and activated microglia (Ito et al., 1998) and cluster of differentiation 68 (CD68), a macrophage-specific lysosomal-associated protein that labels microglia in resting and activated states (da Silva and Gordon, 1999). For anatomic specification in each case, a parallel series of sections was processed with the cresyl violet Nissl stain.

Density of HLA-DR immunoreactivity was assessed in the cortical white matter towards the gyral crown of each region in coronal sections. Multiple photomicrographs were obtained per coronal section, acquired at 4× magnification. At times, over 20 photomicrographs per region were acquired to ensure representative sampling was acquired. If more than one gyrus comprised the region, the cortical white matter was sampled once in each gyrus per section. If the photomicrograph contained gray matter, the image was cropped to ensure that the quantification of activated microglia was limited to the white matter. Photomicrographs containing only white matter were imported into ImageJ software (version 1.51) to measure optical density of HLA-DR immunoreactivity. Optical density (OD) was averaged across photomicrographs and used as a single data point in each region. OD was calculated using the following formula: OD = log (maximum light intensity/mean light intensity), where maximum intensity is 255 for 8-bit images. Images displaying greater concentration and/or size of HLA-DR-positive microglia equated to lower light transmission, resulting in higher optical density values. We have used this method in previous investigations of white matter microglia (Ohm et al., 2019).

### Image Acquisition Parameters and Ratings of White Matter Hyperintensities (WMHs)

Two cases had MRI FLAIR scans available prior to death. MRI scans were acquired on a 3T Siemens TRIO system using a 12-channel birdcage head coil. Imaging was performed at the Northwestern University Center for Translational Imaging (CTI). Visual ratings of WMHs were performed by a behavioral neurologist (CO) using the Cardiovascular Health Study (CHS) visual rating scale, which has been previously validated (Manolio et al., 1994; Liao et al., 1997); the scale is divided into four categories of absent, mild, moderate, or severe WMH based on the convention set by the NACC Uniform Data Set, version 3.0.

### Statistical Analysis

Mean optical densities of white matter activated microglia were compared between groups with a two-way ANOVA and *post hoc* tests. One-way ANOVAs were used to assess for group differences in case characteristics. Data that represented the regional distribution of microglia were not normally distributed. As a result, Spearman correlational analyses were used to determine relationships between age and white matter activated microglia. Significance was set to *p* < 0.05 for all comparisons. Statistical analyses were performed using GraphPad Prism (version 7.0 for Mac, GraphPad Software, La Jolla, CA, USA^2^).

## Results

### Activated Microglia Density Across Aging Trajectories

One-way ANOVAs were used to determine differences in age at death, post-mortem interval (PMI) and brain weight between young, cognitively normal old adults, and SuperAgers. There were significant differences in age at death between young and both elderly groups (*p* < 0.0001) but not between SuperAgers and cognitively normal old adults. There were no significant differences in brain weight or PMI.

**Figure 1:**
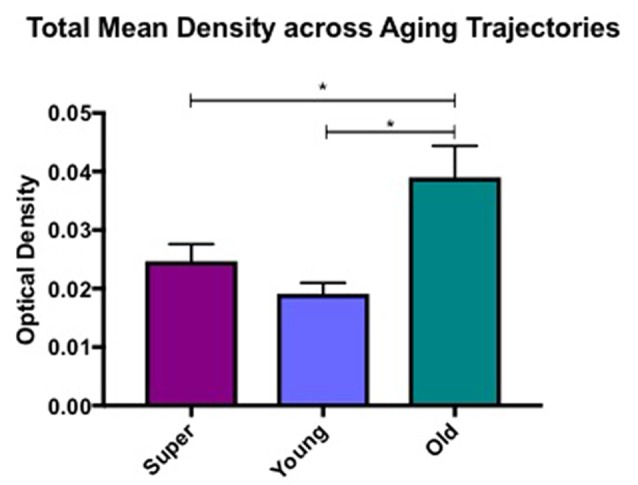
Total mean white matter microglia density per group (regions averaged) is depicted, which shows higher densities in cognitively normal old compared to young and SuperAging cases. * = significant at *p* < 0.05; error bars = standard error.

**Figure 2:**
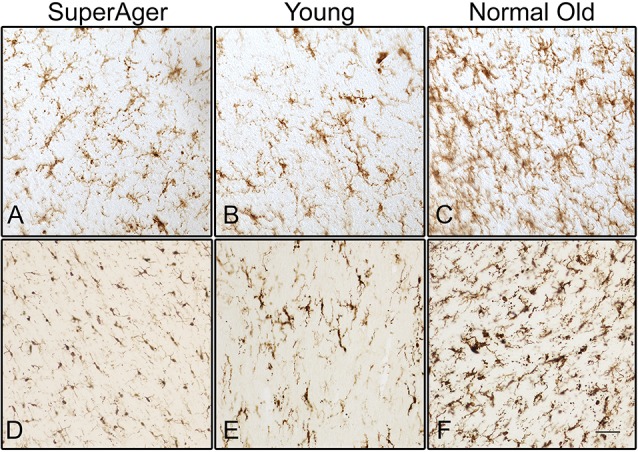
Human leukocyte antigen-D related (HLA-DR)-positive activated microglia are shown in the white matter of the inferior frontal gyrus (IFG) in **(A)** SuperAger (Case 17), **(B)** Young (Case 2), and **(C)** Normal Old (Case 9). Microglia immunoreactive to Iba1 are shown in corpus callosum (CC) in **(D)** SuperAger (Case 16), **(E)** Young (Case 2), and **(F)** Normal Old (Case 10). Photomicrographs were acquired at 20× magnification. Scale bar = 100 μm.

**Figure 3:**
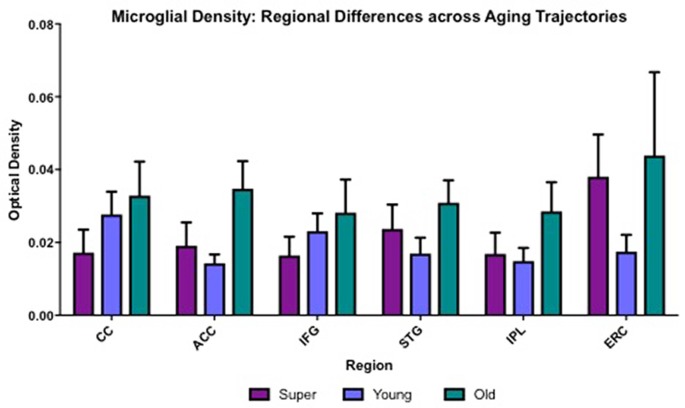
Regional differences in white matter microglia density across three cognitive aging groups are shown. CC, corpus callosum; ACC, anterior cingulate cortex; IFG, inferior frontal gyrus; STG, superior temporal gyrus; IPL, inferior parietal lobule; ERC, entorhinal-hippocampus complex. Error bars = standard error.

Differences in microglia density were analyzed in a two-way ANOVA with *post hoc* Newman-Keuls multiple comparison tests; brain region (CC, ACC, IFG, STG, IPL, and ERC) and aging trajectory group (Young, Old, and SuperAger) were included as independent variables. There was a significant main effect of group on differences in microglia density, *F*_(2,81)_ = 4.87, *p* = 0.01, where the old showed higher overall densities compared to both young (*p* < 0.05) and SuperAgers (*p* < 0.05). There was no difference, however, in overall microglia densities between SuperAgers and the young. Total mean density per group (regions collapsed) is depicted in Figure 1, which shows the main effect of group on density, and Figures 2A–C, which pictorially illustrate differences in densities across groups. The main effect of region on microglia density was not significant, *F*_(5,81)_ = 0.79, *p* = 0.56, nor was there a significant region × group interaction, *F*_(10,81)_ = 0.45, *p* = 0.92. Although there were no significant regional differences across aging trajectories, SuperAgers showed the lowest microglia density in CC and IFG, and nearly equal densities compared to the young in ACC and IPL (see Figure 3). SuperAgers and the Old showed high and comparable densities in ERC; this was particularly striking when compared to young cases. Across cases, HLA-DR staining appeared greater in white matter compared to gray matter, due to larger, denser populations of activated microglia based on examination at high magnification. Cases 2, 10, and 16 were qualitatively evaluated with CD68 and Iba1, which showed densities that followed similar patterns observed with HLA-DR; see Figures 2D–F.

One-tailed Spearman correlational tests were used to determine relationships between age and regional differences in microglia density among young (*N* = 5) and cognitively normal old (*N* = 7) controls. Data in this correlational analysis did not pass tests of normality. There were significant positive relationships between age and microglia density in ACC (*r* = 0.62; *p* = 0.02), STG (*r* = 0.52; *p* = 0.04), and ERC (*r* = 0.56; *p* = 0.048), neuroanatomic regions that are strongly implicated in the pathophysiology of aging.

### MRI FLAIR and White Matter Microglia Density in Two SuperAging Cases

**Figure 4:**
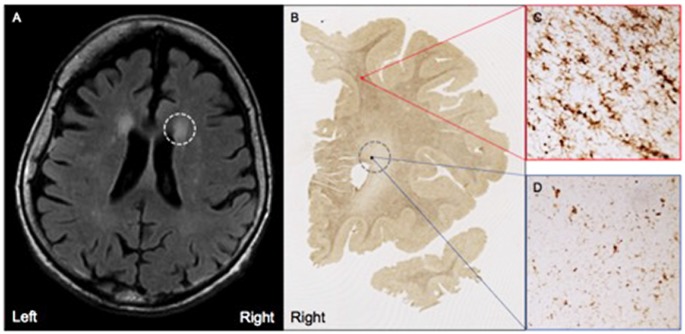
**(A)** Moderate white matter hyperintensities (WMHs) are observed in periventricular regions on T2 FLAIR MRI from Case #17, a SuperAger. **(B)** Representative HLA-DR staining in a whole-hemisphere coronal section from Case #17 at the level of the WMH shown in **(A)**, demarcated by the gray dotted circle. In comparison to regions of white matter without observed WMHs **(C)**, regions with observed white matter hyperintensities **(D)** displayed less HLA-DR-immunopositivity, which was reflected by smaller, less dense populations of activated microglia. Photomicrographs were acquired at 20× magnification.

MRI FLAIR images were available from Case 16 within 8 months prior to death and Case 17 within the month prior to death. Characteristics of both cases are presented in Table 1. Both Case 16 and Case 17’s CHS showed “mild” levels of WMHs, and both cases had prominent hyperintensities in periventricular regions. These specific loci were examined carefully in postmortem serial sections of both Case 16 and 17 to determine whether there were equally prominent microglia densities. Interestingly, periventricular areas in postmortem sections showed sparse appearance of activated microglia; and in fact, case 17, the oldest of the entire sample at age 99, had the overall lowest mean microglia densities (all regions averaged) including the younger sample; see Figure 4.

## Discussion

This study examined age-related changes in microglia density, a marker of neuroinflammation, across multiple cognitive aging trajectories. It is well-known that persistent activation of local immune cells (microglia) in cortical regions of the brain trigger a long-lasting inflammatory response that includes the release of molecules such as proinflammatory cytokines and chemokines, and the enhancement of oxidative stress (Frank-Cannon et al., 2009; Raj et al., 2017). Our understanding of the role of microglia and their neuroinflammatory response in the white matter of the aging human brain, however, is considerably limited. There is also a paucity of information regarding the potential contribution of white matter microglia to the preservation of cognitive function in humans. In this study, we investigated activated microglia in the white matter of postmortem specimens of young and old cognitively normal individuals, and a group of cognitive SuperAgers who demonstrate memory performance at levels commensurate with individuals 30 years their junior. This latter group is particularly unique in that prior findings have shown both *in vivo* anatomic and postmortem pathologic differences, compared to cognitively-average-for-age peers (Harrison et al., 2012; Cook et al., 2017; Rogalski et al., 2018). In a recent series of findings from the first 10 SuperAging cases that came to autopsy, specimens showed variable findings within the spectrum of Alzheimer pathology with neurofibrillary degeneration mostly in the Braak II–III stages in ERC (Rogalski et al., 2018). Interestingly, these areas still contained intact, healthy appearing neurons, with a general lack of neurofibrillary degeneration in cortical regions. The lower-than-expected neuropathologic burden in SuperAgers given their age may suggest one avenue for heightened memory abilities. In the current study, our findings showed levels of activated microglia in white matter of SuperAgers that were more consistent with younger-aged individuals rather than same-aged peers; although no significant regional differences were demonstrated, we showed positive relationships between age and microglia density in white matter underlying the ACC, STG, and ERC, regions of dense connectivity that tend to show an age-related emergence of Alzheimer pathology in the postmortem human brain (Braak and Braak, 1991). The relative absence of white matter activated microglia in SuperAgers suggests yet another interesting substrate that may underlie superior memory abilities despite old age.

It is well established that age is the greatest risk factor for the emergence of Alzheimer pathology. There have been a number of studies that emphasize the link between activated microglia and Alzheimer pathology in cortical gray matter (McGeer et al., 1987; Styren et al., 1990; Block and Hong, 2005; Perry et al., 2010; Serrano-Pozo et al., 2011), but few human studies have addressed activated microglia in underlying white matter. Age-related microglia changes, however, have been demonstrated within white matter tracts in the rhesus monkey. In one early study, microglia activation increased with age in a diffuse fashion within white matter tracts; the extent of activation was related to the degree of cognitive impairment (Sloane et al., 1999). Consistent with our findings in the human, another study restricted to an investigation of the optic nerve in the rhesus monkey showed that both oligodendrocytes and microglia become more numerous with age. The authors observed that microglia became engorged with phagocytosed debris, some of which was recognized as degenerating myelin (Sandell and Peters, 2002). The precise impact of activated microglia in white matter remains unknown, with some possibilities—in addition to myelin loss and dysregulation of chemokine and cytokine production—including important protective and regenerative effects when confronted with injury (Ziebell and Morganti-Kossmann, 2010). Overall, our findings point to the emergence of white matter abnormalities in the course of normal aging, which are likely to contribute to age-related cognitive decline.

The literature is replete with evidence to support the role of microglia in cortical gray matter pathogenesis in a wide range of neurodegenerative diseases including AD (Perry et al., 2010; Serrano-Pozo et al., 2011), but less so in subcortical white matter. Prior findings from our group investigated the density and distribution of activated microglia in the white matter of individuals with postmortem AD and an antemortem clinical diagnosis of primary progressive aphasia (PPA), a dementia syndrome characterized by progressive language impairment (Mesulam, 2003); findings suggested that white matter activated microglia accumulation corresponds to patterns of gray matter atrophy in PPA (Ohm et al., 2019). Although the current study did not investigate microglia density in associated cortical gray matter or focal patterns of age-related atrophy, areas of dense activated microglia were more pronounced in white matter underlying limbic regions within the old cases compared to the young. Additional studies that measure the status of oligodendrocytes, myelin, and axons will be critical to determine the extent and type of pathogens that emerge in white matter across cognitive aging trajectories.

Insights into white matter degeneration across the lifespan have been derived from *in vivo* imaging studies like diffusion tensor imaging (DTI) and novel automated longitudinal tractography (TRACULA; Lee et al., 2015; Storsve et al., 2016). Raj et al. (2017) provided recent evidence of increased microglia-induced neuroinflammation in the white matter of aging and brains of individuals with AD using positron emission tomography (PET) imaging with [^11^C]-(R)-PK11195. CSF-based biomarkers are in the process of being tested for detection of microglia-induced neuroinflammation in white matter to identify disease onset at early stages (Varrone et al., 2015; Zhang, 2015). Prior research has focused on WMHs, evident on T2 FLAIR MRI, as a possible marker for age-related decrease in white matter integrity. While the etiology of WMHs remains unclear, age is one of the only non-refuted risk factors for WMHs (Longstreth et al., 1996), with WMHs present in over 80% of individuals over age 80 (de Leeuw et al., 2001). Generally, WMHs are believed to represent small vessel disease, given the association with vascular risk factors (Breteler et al., 1994; Jeerakathil et al., 2004), and pathological studies showing lipohyalinosis, vessel ectasia, demyelination, and decreased fidelity of the blood-brain barrier in normal elderly (Awad et al., 1986; Pantoni and Garcia, 1997). Age-related pathologic correlates of WMHs, and their contribution to cognitive function, however, has not been well studied in the human brain. An ancillary goal of this study was to determine whether WMH burden evident on *in vivo* scans corresponded to areas of heightened microglia density. Qualitatively, we highlighted two autopsied cases of SuperAgers with MRI FLAIR available during life, that show prominent hyperintensities in periventricular regions but a sparse appearance of activated microglia in corresponding sections—suggesting a dissociation between *in vivo* WMH and postmortem microglia burden. Other than the obviously small sample size, one possible reason for this dissociation is that there are inherent pathologic differences between periventricular vs. subcortical signal abnormalities (Schmidt et al., 2011). Studies examining larger cohorts are required to determine whether this finding is universal or a characteristic of the unique sub-population of SuperAgers examined in this study.

Future studies will focus on investigating the relationship between microglia activation in white matter and other age-related pathologic markers across the cognitive lifespan. We conclude from this study that microglia likely activate in response to age-related pathologic changes; this phenomenon appears to be largely spared in SuperAgers, individuals who avoid typically age-related decline in episodic memory performance compared to their peers. Understanding the mechanisms that contribute to white matter integrity across the lifespan offers insights into the preservation of cognition in the old and will be important for identifying potential neuroinflammatory markers associated with age-related changes in the human brain.

## Author Contributions

TG contributed to the design and conceptualization of the study and drafting the manuscript. GK, KB, AG, RS, and DO contributed to the generation, acquisition, and interpretation of data. CO contributed to the interpretation of MRI data. SW contributed to the determination of clinical diagnosis and revising the manuscript for intellectual content. AR contributed to the statistical analysis of data. EB contributed to pathologic diagnosis and revising the manuscript for intellectual content. CG, ER, and M-MM contributed to the design and conceptualization of the study, interpretation of data, and drafting and finalizing the manuscript.

## Conflict of Interest Statement

The authors declare that the research was conducted in the absence of any commercial or financial relationships that could be construed as a potential conflict of interest.

## Footnotes

^1^http://www.wma.net/e/policy/17-c_e.html

^2^www.graphpad.com
